# Immune-Related Long Non-Coding RNA Signatures for Tongue Squamous Cell Carcinoma

**DOI:** 10.3390/curroncol30050363

**Published:** 2023-05-08

**Authors:** Daniel Hu, Diana V. Messadi

**Affiliations:** 1 School of Dentistry, University of California, Los Angeles, CA 90095-1668, USA; 2 Jonsson Comprehensive Cancer Center, University of California, Los Angeles, CA 90095-1668, USA

**Keywords:** tongue squamous cell carcinoma, immune-related genes, lncRNA, survival analysis, prognosis

## Abstract

Background: Tongue squamous cell carcinoma (TSCC) represents one of the major subsets of head and neck cancer, which is characterized by unfavorable prognosis, frequent lymph node metastasis, and high mortality rate. The molecular events regulating tongue tumorigenesis remain elusive. In this study, we aimed to identify and evaluate immune-related long non-coding RNAs (lncRNAs) as prognostic biomarkers in TSCC. Methods: The lncRNA expression data for TSCC were obtained from The Cancer Genome Atlas (TCGA) and the immune-related genes were downloaded from the Immunology Database and Analysis Portal (ImmPort). Pearson correlation analysis was performed to identify immune-related lncRNAs. The TCGA TSCC patient cohort was randomly divided into training and testing cohorts. In the training cohort, univariate and multivariate Cox regression analyses were used to determining key immune-related lncRNAs, which were then validated through Cox regression analysis, principal component analysis (PCA), and receiver operating characteristic (ROC) analysis in the testing cohort. Results: Six immune-related signature lncRNAs (MIR4713HG, AC104088.1, LINC00534, NAALADL2-AS2, AC083967.1, FNDC1-IT1) were found to have prognostic value in TSCC. Multivariate and univariate cox regression analyses showed that the risk score based on our six-lncRNA model, when compared to other clinicopathological factors (age, gender, stage, N, T), was an important indicator of survival rate. In addition, Kaplan–Meier survival analysis demonstrated significantly higher overall survival in the low-risk patient group than the high-risk patient group within both training and testing cohorts. The ROC analysis indicated that the AUCs for 5-year overall survival were 0.790, 0.691, and 0.721, respectively, for training, testing, and entire cohorts. Finally, PCA analysis demonstrated that the high-risk and low-risk patient groups presented significant deviation regarding their immune status. Conclusions: A prognostic model based on six immune-related signature lncRNAs was established. This six-lncRNA prognostic model has clinical significance and may be helpful in the development of personalized immunotherapy strategies.

## 1. Introduction

Tongue squamous cell carcinoma (TSCC) is the most common malignancy of the oral cavity [1,2]. A recent study suggested that about 45% of all oral cavity cancers were TSCCs [3]. TSCC generally affects the older age group of patients (e.g., 60 to 80 years of age) who have been exposed to tobacco and/or drinking for certain period of time. However, recent studies indicate that there has been an increasing incidence of TSCC in the younger age group [4,5,6]. TSCC in young age patients may not necessarily be due to direct exposure to smoking and drinking alcohol but might be caused by human papilloma virus infection [7]. Etiologically, it represents a distinct disease entity from TSCC in older patients.

According to the National Cancer Institute Cancer Statistics, an estimated 17,860 new cases of TSCC and 2790 related deaths are projected to occur in the United States in 2022 [8]. The rate of new cases of tongue cancer is 3.6 per 100,000 men and women annually, with a death rate of 0.7 per 100,000 men and women per year. These rates are based on age-adjusted data from 2015–2019 cases and 2016–2020 deaths. In 2017–2019, approximately 0.4% of men and women were diagnosed with tongue cancer at some point in their lifetime. TSCC is generally an aggressive cancer with a relatively poor prognosis [2,9], and with a 5-year relative survival rate of 68.8% in the United States between 2012–2018 [8]. Despite advancements in treatment options, the survival rates for TSCC patients have remained relatively unchanged over the past few decades. Factors that contribute to the poor prognosis of TSCC include late diagnosis, high rates of metastasis, and the tendency for tumors to be aggressive and resistant to treatment.

Early detection plays a critical role in the survival of TSCC patients, as the prognosis and five-year survival rate improve significantly with early diagnosis. Among TSCC patients, 29% are diagnosed at the local stage where the tumor is confined to the primary site, 53% are diagnosed at the regional stage where the tumor has spread to regional lymph nodes, 15% are diagnosed at the distant stage where the tumor has metastasized, and the remaining 4% are diagnosed with an unknown stage [8]. Based on data from the National Cancer Institute Cancer Statistics, the 5-year relative survival rates for localized, regional, distant, and unknown stage tongue cancer are 84.2%, 69.8%, 40.7%, and 53.0%, respectively [8]. These rates highlight the importance of early detection and timely treatment, as patients with localized tumors have significantly better survival rates compared to those with advanced disease. Therefore, regular screening and monitoring of high-risk individuals, such as those with a history of smoking and heavy alcohol consumption, can help with early detection and improve patient outcomes.

The clinicopathologic diagnosis of TSCC does not always provide accurate predictions for clinical outcomes, highlighting the need for reliable prognostic biomarkers to guide treatment strategies. Recent advancements in molecular pathology have introduced thousands of tumor biomarkers associated with the progression and/or prognosis of various cancers, many of which have been evaluated for their prognostic value in OTSCC. However, despite the presentation of several promising biomarkers that could provide additional value to traditional factors such as stage, tumor grade, and depth of invasion, none have been approved for daily clinical use [10].

The aim of this study was to investigate the expression of immune-related long non-coding RNAs (lncRNAs) in patients with TSCC and identify a panel of lncRNAs that could serve as prognostic biomarkers for TSCC. LncRNAs are a type of RNA that are longer than 200 nucleotides and do not encode proteins [11]. Over the past decade, studies have revealed that lncRNAs have diverse roles in gene regulation, including modulating chromatin function, regulating nuclear bodies, and altering mRNA stability and translation. These functions can ultimately affect gene expression in various signaling pathways, such as those involved in immune responses and cancer [12]. While lncRNAs have been linked to head and neck cancer, including TSCC [13,14], little is known about their clinical applications or functional roles of immune-related lncRNAs in TSCC.

## 2. Materials and Methods

### 2.1. Workflow

**Figure 1:**
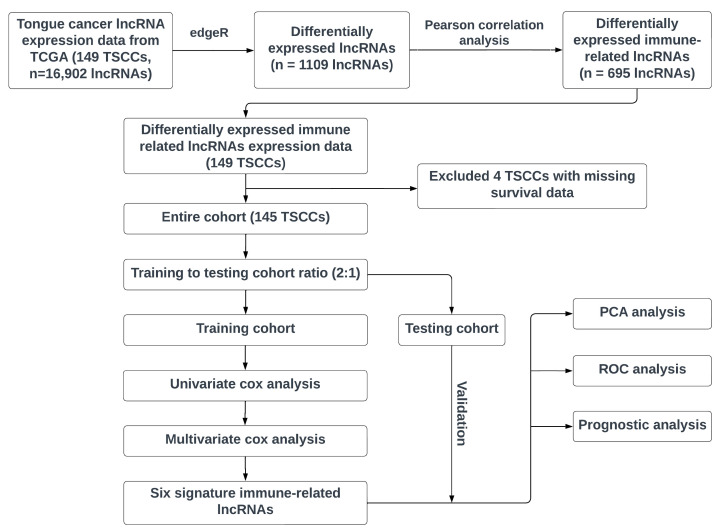
The workflow of our study design to discover an immune-related signature lncRNA model. TSCC: Tongue squamous cell carcinoma. TCGA: The Cancer Genome Atlas. The lncRNA profiling data of TSCC patients were extracted from the TCGA database and lncRNAs at significantly differential levels between TSCCs and normal controls were identified. Subsequently, the Pearson correlation analysis was performed to identify differentially expressed immune-related lncRNAs. Next, the TSCC patients who have meaningful survival data (*n* = 145, excluding 4 TSCC subjects with missing survival data) were randomly divided into training cohort and testing cohort (training to testing cohort ratio: ~2:1). Both univariate and multivariate cox regression analyses were performed to build the signature lncRNA model. Finally, the testing cohort was used to validate the efficacy of our developed lncRNA model, and subsequent prognosis analysis, ROC analysis, and PCA were performed to evaluate the signature lncRNA model.

Figure 1 illustrates our workflow in developing an immune-related signature lncRNA model for TSCC prognosis. First, we extracted the lncRNA profiling data of TSCC patients from the TCGA, a landmark cancer genomics program with >20,000 primary cancer and matched normal samples spanning a large number of cancer types, and identified the lncRNAs at significantly differential levels in TSCCs. Subsequently, the Pearson correlation analysis was performed to identify differentially expressed immune-related lncRNAs. Next, the TSCC patients who have meaningful survival data (*n* = 145, excluding 4 TSCC patients with missing survival data) were randomly separated into training and testing cohorts (training to testing cohort ratio ~2:1). Both univariate and multivariate cox regression analyses were performed to build the signature lncRNA model. Finally, the testing cohort was used to validate the efficacy of our developed lncRNA model, and subsequent prognosis analysis, receiver operating characteristic (ROC) analysis, and PCA were performed to evaluate the signature lncRNA model.

### 2.2. Data Resource

As previously stated, our data were downloaded from the TCGA’s GDC portal where we extracted the transcriptome profiling data for 149 tongue cancer subjects from the TCGA head and neck squamous carcinoma project. The lncRNA expression levels were extracted and the list of immune genes was imported from the Immunology Database and Analysis Portal.

### 2.3. Immune-Related lncRNAs

The downloaded gene expression and clinical data from the TCGA were organized and merged so that each patient’s transcriptome data corresponded to their clinicopathological data using Perl. Since this study was based on the premise of discovering key immune-related lncRNAs with prognostic value, patients with either unknown survival time or survival times of less than 30 days were removed. We then used the edgeR package in the R program (version 4.2.0) to filter out significantly differentially expressed lncRNAs between the cancer and normal samples, with a threshold of FDR < 0.05 and |log_2_FC| ≥ 1 [15]. With the list of both immune genes from the ImmPort and significantly differentially expressed lncRNAs, we conducted the Pearson correlation coefficient analysis, with a threshold of *p* < 0.001 and |R| > 0.4, to extract differentially expressed immune-related lncRNAs.

### 2.4. Construction of the Immune-Related lncRNA Prognostic Model

To identify potentially valuable lncRNAs for TSCC prognosis, we utilized the “survival” package in the R 4.2.3 to conduct univariate Cox regression analysis on the immune-related lncRNAs obtained. This analysis was performed to identify key lncRNAs (*p* < 0.01), which were then further screened using a stepwise multivariate Cox regression model. Based on the expression level of the lncRNAs and the regression coefficient β of the weighted linear combination in the multivariate analysis, the risk score of each patient was calculated using the following formula: Risk score = βgene_1_ × exprgene_1_ + βgene_2_ × exprgene_2_ + … + βgene_n_ × exprgene_n_ (βgene_n_: coefficient; exprgene_n_: expression level). Using the median risk score, the patients were divided into low-risk and high-risk groups.

### 2.5. Application and Validation of the Risk Score Model

The Kaplan–Meier curves were created to estimate the survival capability for the low- and high-risk groups being tested. Independent prognostic analysis was then carried out through univariate and multivariate cox proportional hazards regression analysis. Subsequently, the receiver operating characteristics (ROC) analyses were performed to compute the area under curve (AUC), verify our model, and to evaluate the accuracy of our model in comparison with other clinicopathological factors.

### 2.6. Principal Components Analysis (PCA)

With the R packages “limma” and “scatterplot3d”, the PCA analysis was performed to visualize and compare the predictive power of all immune-related lncRNAs or the six immune-related signature lncRNAs.

## 3. Results

### 3.1. Data Processing and Differential Expression Analysis

**Figure 2:**
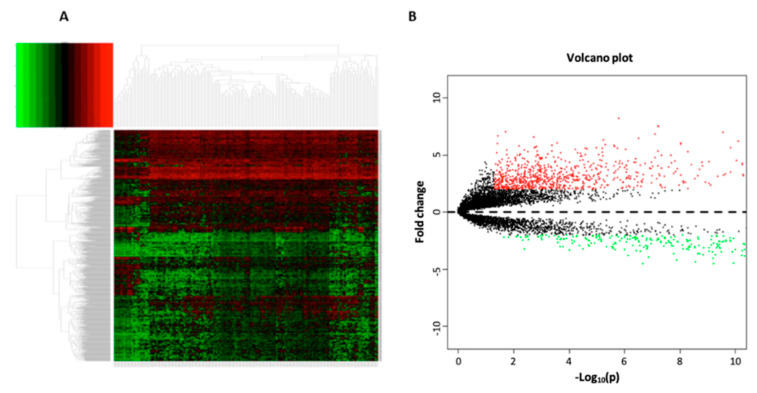
Heat map (**A**) and volcano plot (**B**) of differentially expressed lncRNAs between TSCC (*n* = 149) and normal (*n* = 15) tissue samples. The heat map indicates the lncRNAs at significantly differential levels (adjusted *p* < 0.05, |log_2_FC| ≥ 1, FC: fold change) between TSCC and normal controls, whereas the volcano plot presents the FC and *p* values of all lncRNAs between TSCCs and normal controls. In the volcano plot, red dots represent significantly upregulated lncRNAs, whereas green dots represent significantly downregulated lncRNAs (adjusted *p* < 0.05, |log_2_FC| ≥ 1). The black dots represent lncRNAs with non-significant changes in TSCCs (Figure 2B).

The clinicopathological and transcriptomic data of 149 TSCC subjects were downloaded from the TCGA-HNSC project initially. A total of 16,902 lncRNAs were extracted from the complete transcriptome profiles, and through differential expression analysis with the edgeR package, 1108 lncRNAs were found to be significantly differentially expressed between the TSCC and normal tissues (adjusted *p* < 0.05 and |log2FC| ≥ 1). The heat map and volcano plot of significantly changed lncRNAs in TSCCs are shown in Figure 2. Using the list of immune related genes (*n* = 2483) from the ImmPort, the expression data of 1502 immune-related genes was extracted from the TSCC dataset, and then, through Pearson correlation analysis of the expression between 1108 lncRNAs and 1502 immune-related genes (mRNAs), 695 differentially expressed, immune-related lncRNAs were obtained (*p* < 0.001 and |R| > 0.4). These immune-related lncRNAs had either positive or negative modulation on the expression of the immune-related genes.

### 3.2. Identification of Immune-Related Signature lncRNAs and Construction of Six-lncRNA Prognostic Model

Patients with missing survival data or with survival times of less than 30 days were excluded from the model building. Therefore, 145 TSCC patients were included for subsequent analysis in this study. The demographic data of the 145 TSCC patients, including age, gender, and TNM stage, are shown in Table 1.

Using the R software, the TSCC subjects were randomly divided into training and testing cohorts by a ratio of 2:1. In the training cohort, univariate analysis was performed on the expression profiles of the 695 differentially expressed, immune-related lncRNAs, resulting in 10 immune-related signature lncRNAs that had potential prognostic value (Table 2).

For the ten immune-related signature lncRNAs, stepwise multivariate Cox regression analysis was performed to further screen the immune-related lncRNAs, resulting in six immune-related signature lncRNAs (Table 3). These six signature lncRNAs were then used to construct the prognostic model for TSCC. Using the following formula described in Section 2.4, the risk score was calculated for each patient. The TSCC patients were then divided into low-risk and high-risk groups based on the median risk score. The *p* value for each of the six lncRNA genes is <0.05, indicating that these genes are statistically significant.

### 3.3. Validation of Six Immune-Related lncRNA Prognostic Model

**Figure 3:**
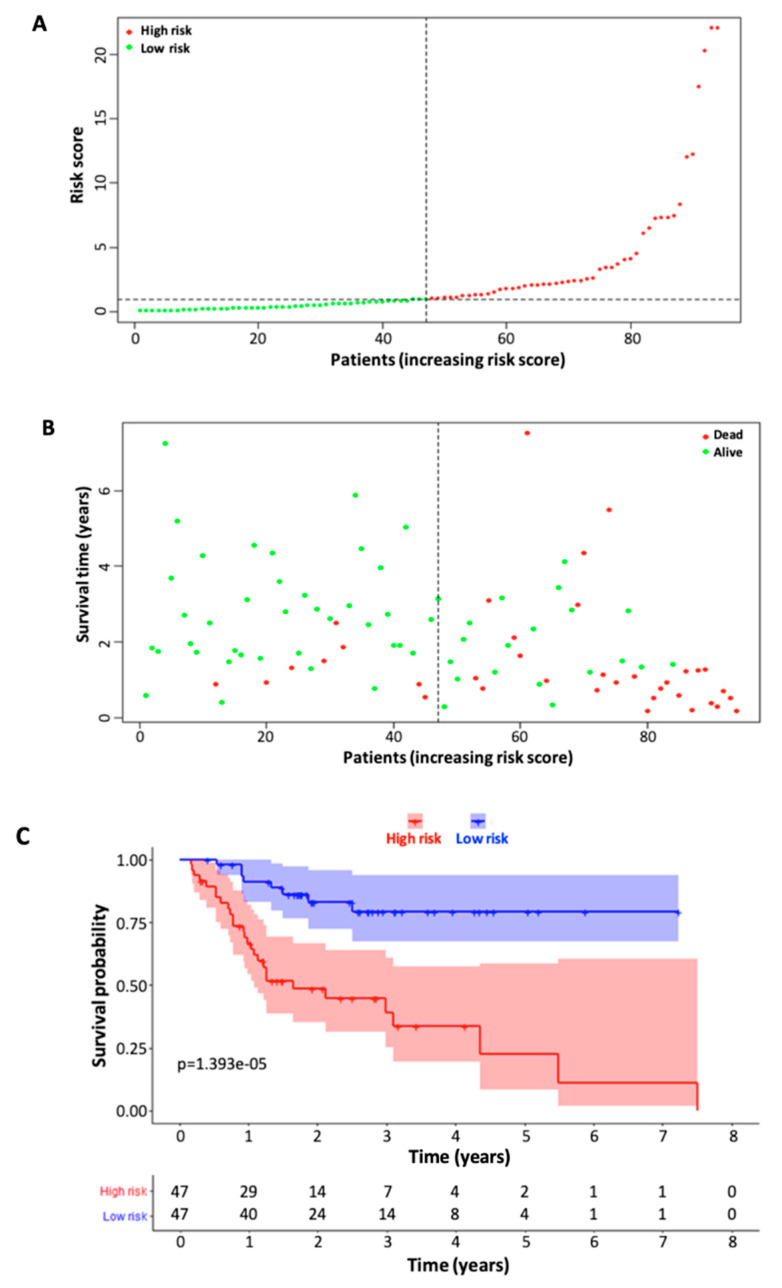
Construction of the six-immune-related-lncRNA signature model through the training cohort (*n* = 94). (**A**) Distribution of the risk scores of the TSCC patients in the training cohort. The risk scores were calculated based on the six signature lncRNAs using the following formula: Risk score = βgene_1_ × exprgene_1_ + βgene_2_ × exprgene_2_ + … + βgene_n_ × exprgene_n_ (βgene_n_ being the coefficient and exprgene_n_ being the expression level). The risk score was calculated for each TSCC patient and then the patients were divided into high- and low-risk groups according to the median risk score. (**B**) Survival status and time of the patients in high- and low-risk groups (training cohort). (**C**) Kaplan–Meier survival curves of the patients in high-risk and low-risk patient groups.

**Figure 4:**
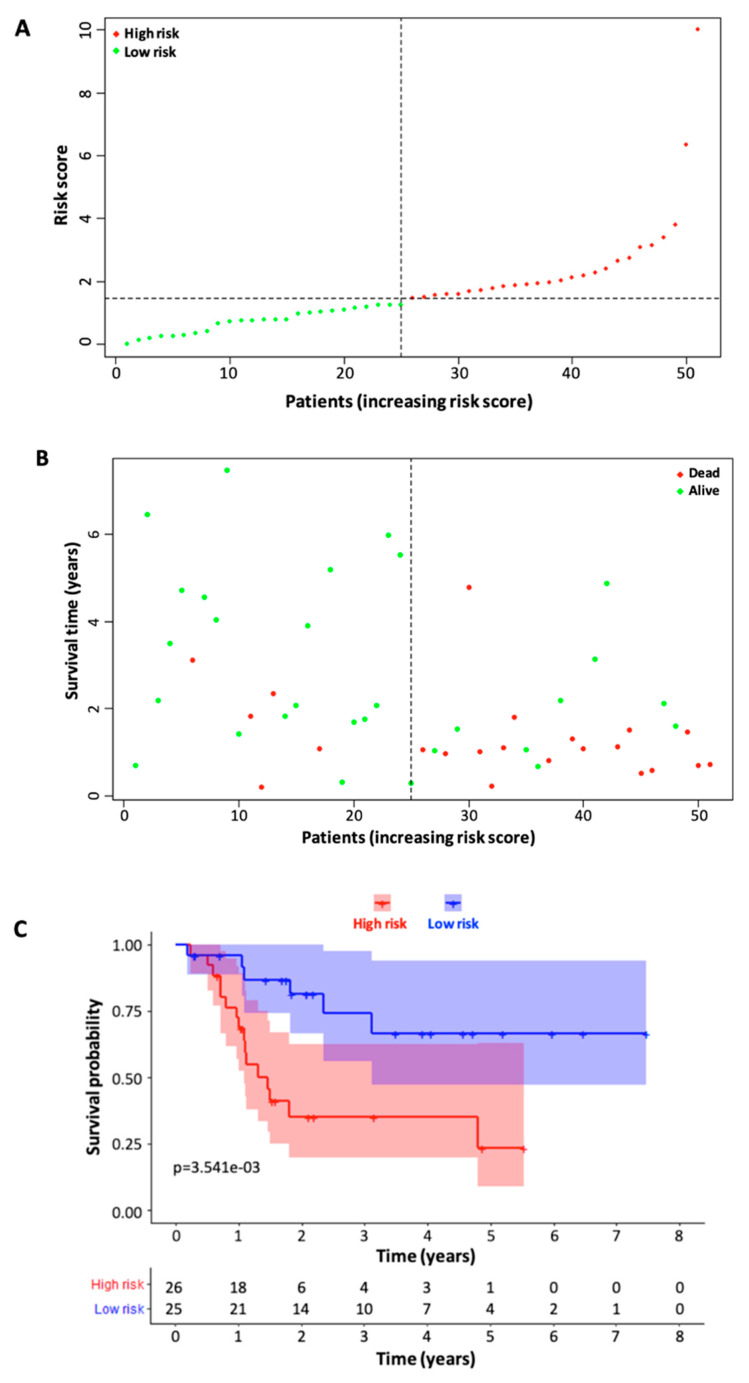
Validation of the six-immune-related-lncRNA signature model through the testing cohort (*n* = 51). (**A**) Distribution of the risk scores for the patients in the testing cohort. (**B**) Survival status and time of the patients in high- and low-risk groups (testing cohort). (**C**) Kaplan–Meier survival curves of the patients in high- and low-risk patient groups. Refer to Figure 3 legend for the details of risk score calculation and dividing of low- and high-risk patient groups based on median risk score.

Once the risk scores for the training cohort patients were all calculated using the formula above, the median risk score was then calculated and used to separate patients into high-risk and low-risk groups (Figure 3A). The distribution of the survival status between high-risk and low-risk patient groups is visualized in Figure 3B. The Kaplan–Meier survival curve demonstrated a relatively clear difference in patient survival probability between the high-risk and low-risk groups (Figure 3C). We then validated the model with the testing cohort by performing the same type of analysis, and the data analysis showed similar results to those of the training cohort (Figure 4).

### 3.4. ROC Analysis

**Figure 5:**
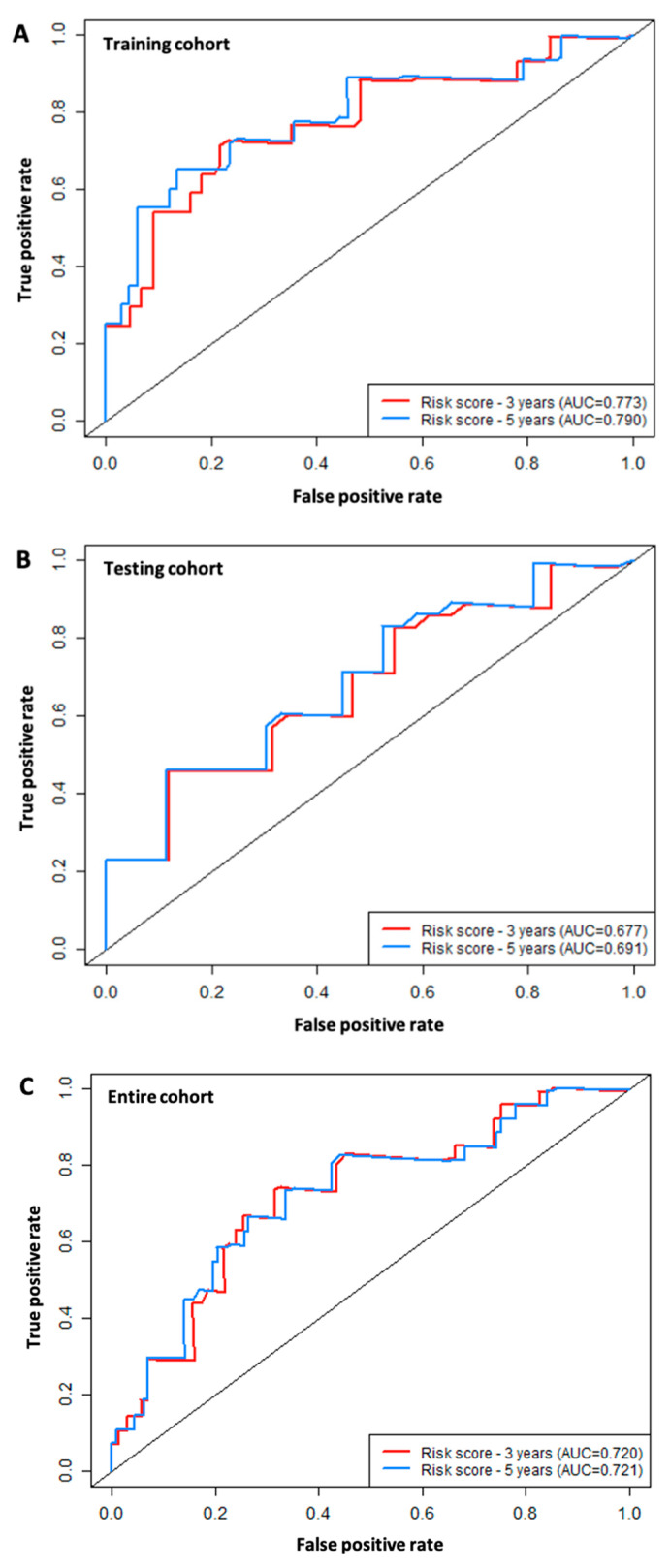
ROC analysis of the six-immune-related-lncRNA signature model. (**A**) 3-year and 5-year survival ROC curves for the training cohort (*n* = 94). (**B**) 3-year and 5-year survival ROC curves for the testing cohort (*n* = 51). (**C**) 3-year and 5-year survival ROC curves for the entire cohort (*n* = 125). AUC: Area under the curve.

The 3-year and 5-year ROC curves for the six immune-related signature lncRNAs based on the data of the training cohort were computed to evaluate the performance of the six-lncRNA model, resulting in AUC values of 0.773 and 0.795, respectively (Figure 5A). The same analysis was carried out for both the testing cohort (Figure 5B), which resulted in the 3-year and 5-year AUC values of 0.677 and 0.754, respectively, and the entire cohort, which resulted in the AUC values of 0.677 and 0.754, respectively (Figure 5C).

### 3.5. Comparison of the Six-lncRNA Model with Other Clinicopathological Parameters in TSCC

**Figure 6:**
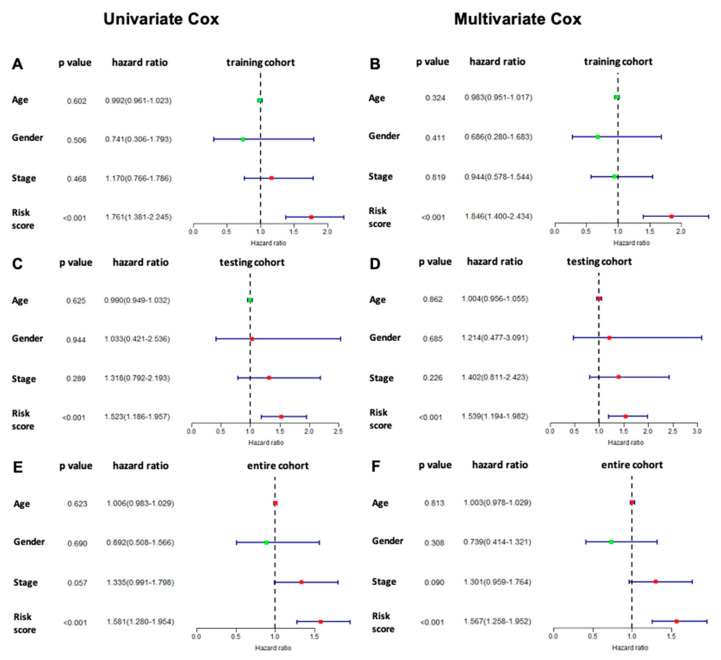
Univariate and multivariate Cox regression analysis for evaluating six-lncRNA signature model vs. individual clinicopathological factors. (**A**) Univariate and (**B**) multivariate Cox regression analyses of the six-lncRNA model in the training cohort. (**C**) Univariate and (**D**) multivariate Cox regression analyses of the six-lncRNA model in the testing cohort. (**E**) Univariate and (**F**) multivariate Cox regression analyses of the six-lncRNA model in the entire cohort. Univariate cox regression calculates the hazard ratio between a variable and the survival of a TSCC patient independent of all other variables, whereas multivariate cox regression takes into account the other variable present when calculating the hazard ratio. Higher median hazard ratios with less variation of our six-lncRNA model were observed when compared to the clinicopathological factors, age, gender, and cancer stage.

We performed both univariate and multivariate Cox regression analyses to determine if our model can be independently used for prognosis in TSCC. We included other clinicopathological characteristics such as age, gender, and stage (Figure 6) for comparison. Both univariate and multivariate Cox regression analyses indicated higher median hazard ratios with less variation of our six-lncRNA model when compared to the clinicopathological factors (age, gender, and stage). The results suggested that our six-lncRNA risk score model was a prognostic predictor independent of age, gender, and stage.

### 3.6. Prognostic Value of the Six Immune-Related lncRNA Model

**Figure 7:**
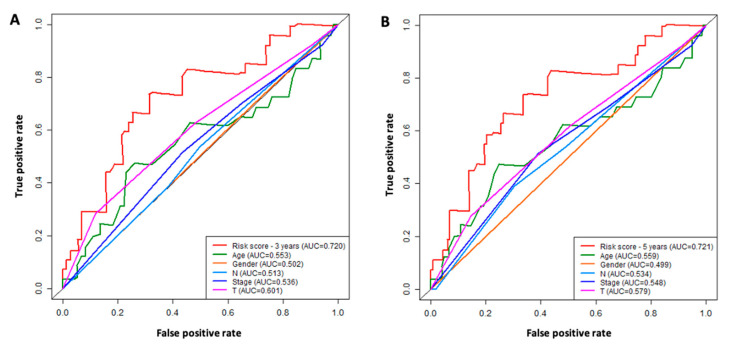
ROC analysis for the comparison of six-lncRNA signature model with clinicopathological factors. (**A**) 3-year survival ROC curves for the risk score (based on six-lncRNA model) and clinicopathological factors (age, gender, stage, lymph node N, tumor size T). (**B**) 5-year survival ROC curves for the risk score (based on six-lncRNA model) and clinicopathological factors (age, gender, stage, lymph node N, tumor size T). The six-lncRNA signature model shows the highest AUC values.

The ROC analysis was performed to determine the clinical significance of the six lncRNA signatures in comparison with other clinicopathological factors such as age, gender, stage, T, and N (Figure 7). The ROC curves indicate that our six-lncRNA model had the highest AUC value when compared to the other factors for both 3-year (Figure 7A) and 5-year overall survival (Figure 7B).

### 3.7. Immune Status Associated with the Six Signature lncRNAs

**Figure 8:**
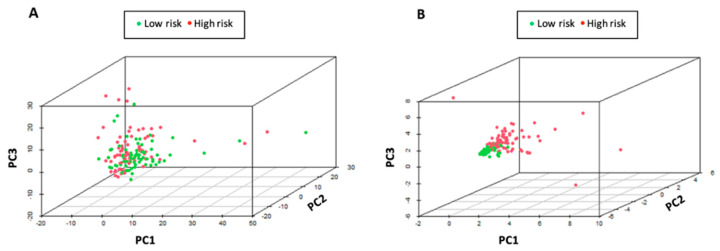
Principal component analysis (PCA). (**A**) PCA between high- and low-risk patient groups based on the expression profiles of all immune-related lncRNAs. (**B**) PCA between high- and low-risk patient groups based on the expression profiles of six immune-related signature lncRNAs. High- and low-risk patient groups were well separated based on the expression profiles of the six immune-related signature lncRNAs.

PCA analysis was performed to show the difference between the high- and low-risk groups based on the expression profiles of six immune-related signature lncRNAs or all immune-related lncRNAs (Figure 8). Clear separation between the two patient groups was demonstrated based on the expression profile of the six immune-related signature lncRNAs. The two patient groups tended to be distributed in different directions, indicating that there was a significant difference in the immune status between high-risk and low-risk patient groups.

## 4. Discussion

lncRNAs represent a class of RNA molecules that are longer than 200 nucleotides and lack the ability to encode proteins. Once thought to be “junk” RNA, lncRNAs have been found to play important roles in a number of biological processes, including transcriptional regulation, RNA processing, post-transcriptional regulation, and protein synthesis, through various mechanisms, such as chromatin remodeling, transcriptional activation or repression, mRNA splicing, and protein interaction [16]. Moreover, lncRNAs have been implicated in diverse biological functions, such as cell proliferation, differentiation, apoptosis, and migration, as well as in development and aging [16,17]. One of the key functions of lncRNAs is their ability to interact with chromatin and regulate gene expression through chromatin remodeling and/or epigenetic modifications. For example, the lncRNA X-inactive specific transcript (XIST) plays a crucial role in X-chromosome inactivation in females by recruiting chromatin modifiers to silence gene expression on one of the two X chromosomes [18]. LncRNAs also have the ability to modulate the activity of other RNA species, including mRNAs and miRNAs, through various mechanisms such as competing for miRNA binding or serving as decoys to sequester proteins and prevent their interaction with other RNAs [19].

The dysregulation of lncRNA expression has been implicated in the pathogenesis of various diseases, including cancer. Many lncRNAs have been found to be differentially expressed in various types of cancer and are thought to be involved in tumorigenesis and cancer progression [20]. For example, HOTAIR (HOX transcript antisense RNA), a lncRNA that is overexpressed in breast, liver, and colorectal cancers, has been shown to promote tumor growth, metastasis, and drug resistance through various mechanisms, including the epigenetic silencing of tumor suppressor genes [21].

Numerous studies have provided insights into the mechanisms underlying the functions of lncRNAs in cancer. LncRNAs have been found to regulate gene expression at various levels, including chromatin remodeling, transcriptional regulation, post-transcriptional regulation, and epigenetic modification [17]. LncRNAs can interact with DNA, RNA, and proteins to exert their regulatory functions. For example, HOTAIR has been shown to interact with polycomb repressive complex 2 (PRC2) and lysine-specific demethylase 1 (LSD1) to regulate gene expression by modulating histone methylation and acetylation [22].

LncRNAs can regulate the expression and activity of oncogenes and tumor suppressor genes in cancer cells. For example, the lncRNA MALAT1 (metastasis-associated lung adenocarcinoma transcript 1) is overexpressed in various types of cancer and involved in cancer cell proliferation, migration, and invasion via promoting the expression of oncogenes such as TGF-β1 [23]. Similarly, the lncRNA HULC (highly upregulated in liver cancer) is overexpressed in hepatocellular carcinoma and promotes liver cancer growth through increasing the expression of the HMGA2 oncogene via sequestration of the microRNA-186 [24]. In addition, lncRNA CCAT1-L, which is transcribed specifically in human colorectal cancers, plays an important role in MYC transcriptional regulation and promotes long-range chromatin looping. Knockdown of CCAT1-L reduces long-range interactions between the MYC promoter and its enhancers. These results suggested an important role of CCAT1-L in gene regulation at the MYC locus in colon cancer [25]. On the other hand, the lncRNA PANDA (p21-associated noncoding RNA DNA damage-activated) inhibits the expression of the oncogene MYC and promotes the expression of the tumor suppressor p21, thereby inhibiting cancer cell proliferation [26].

In addition, lncRNAs may regulate the activity of signaling pathways involved in cancer pathogenesis, such as the Wnt/β-catenin, PI3K/Akt/mTOR, and MAPK/ERK pathways. For instance, the lncRNA H19 has been shown to promote tumor growth and metastasis by activating the Wnt/β-catenin signaling pathway in several types of cancer [27]. Similarly, the lncRNA MALAT1 has been shown to promote proliferation and metastasis of hepatocellular carcinoma cells by activating the MAPK/ERK pathway [28].

lncRNAs can serve as potential biomarkers for cancer diagnosis, prognosis, and therapy. Several lncRNAs have been shown to be associated with clinical outcomes, such as tumor stage, metastasis, recurrence, and survival, in different cancer types [29]. For instance, the lncRNA prostate cancer-associated transcript 1 (PCAT-1) is upregulated in prostate cancer and associated with poor prognosis and tumor progression [30]. LncRNAs have also emerged as promising therapeutic targets for cancer treatment. LncRNA-based therapeutics can be designed to target specific lncRNAs involved in cancer pathogenesis or to modulate their expression levels or activity. For instance, a peptide nucleic acid- based approach has been used to block the ability of lncRNA HOTAIR to interact with EZH2 and subsequently inhibit HOTAIR-EZH2 activity and resensitize resistant ovarian tumors to chemotherapy [31].

Furthermore, recent studies have shown that immune-related lncRNAs may be used to predict cancer patients’ survival and response to immunotherapy [32,33]. This highlights the potential of immune-related lncRNAs as prognostic biomarkers or therapeutic targets for clinical applications in cancer. Researchers have identified immune-related lncRNAs in various cancer types, including breast cancer [34], hepatocellular carcinoma [35], colorectal carcinoma [36], and renal cancer [37]. However, few studies have explored the clinical significance of immune-related lncRNAs in TSCC. As tongue cancer is one of the deadliest forms of oral cavity cancer, it is crucial to develop effective prognostic biomarkers for predicting malignancy behavior and response to treatments such as immunotherapy. Therefore, this study aimed to identify an effective immune-related lncRNA signature model for TSCC prognosis by performing a bioinformatic analysis of lncRNA gene expression data from TSCC patients.

Through the use of univariate Cox regression analysis and multivariate Cox regression analysis of the lncRNA gene expression data of TSCC patients, we were able to identify six immune-related lncRNAs—MIR4713HG, AC104088.1, LINC00534, NAALADL2-AS2, AC083967.1, and FNDC1-IT1—as signature biomarkers for TSCC. Among the six lncRNA biomarkers, AC083967.1 and MIR4713HG were found to be of significant prognostic value in oral squamous cell carcinoma in previous studies, corroborating our results [38,39]. In fact, lncRNA MIR4713HG acted as a pro-tumor factor facilitating cell proliferation and metastasis of TSCC via the let-7c-5p/TMC7 signaling pathway, suggesting MIR4713HG might serve as a promising therapeutic target in TSCC [39]. To the best of our knowledge, the other four lncRNA biomarkers have not been studied in TSCC. However, lncRNA AC104088.1 was significantly overexpressed in non-small cell lung cancer (NSCLC) and suggested as a prognostic biomarker for NSCLC [40]. The expression level of lncRNA LINC00534 was found to be significantly increased in the serum samples of breast cancer patients [41] and also in lung squamous cell carcinoma tissues [42]. LncRNA FNDC1-IT1 was significantly overexpressed in breast squamous cell carcinoma and lung adenocarcinoma tissues and showed prognostic values predicting overall survival [43,44].

Studies also revealed significant upregulation of LncRNA NAALADL2-AS2 in metastatic castration-resistant prostate cancer (mCRPC) and demonstrated NAALADL2-AS2 as a biomarkers of response measure for survival in patients with mCRPC [45]. Androgen regulated lncRNA NAALADL2-AS2 promoted the survival of prostate cancer cells, and transcriptome and pathway analyses revealed that NAALADL2-AS2 modulated the expression of genes involved with cell cycle control and glycogen metabolism [46]. In addition, upregulation of NAALADL2-AS2 was found to be highly dependent on Epstein–Barr virus nuclear antigen 2 (EBNA2) [47].

Based on the six immune-related signature lncRNAs, we constructed a risk score model for the prognosis of TSCC s. To verify our model, the Kaplan Meier survival curves were utilized to illustrate the survival capability of the high- and low-risk patient groups. The *p*-value of the Kaplan Meier survival curve was 1.393 × 10^−5^ indicating a strong correlation between our risk score model and the survival outcome of TSCC patients. We further verified our lncRNA prognostic model through the testing cohort. In our ROC analysis, the AUC value for 3-year ROC of the training and testing cohorts were 0.773 and 0.677, respectively, demonstrating the proficient accuracy of the model. Univariate and multivariate Cox regression analysis on the training cohort, testing cohort, and entire cohort, demonstrated that our risk score model based on the six lncRNA biomarkers can be used as an independent prognostic factor. Further ROC analysis demonstrated that our six-lncRNA model had a significantly greater AUC in comparison to other clinicopathological characteristics such as age, gender, stage, N, and T. Lastly, PCA analysis demonstrated that our six-lncRNA predictive model could classify TSCC patients into different risk groups based on their immune status.

Our study had one limitation, mainly, that the six immune-related signature lncRNAs were only tested on the patient samples from the TCGA database. Further validation studies are warranted to confirm the clinical utility of our developed lncRNA model. Moreover, in vitro and in vivo experiments would be needed to elucidate the molecular mechanisms of the six immune-related signature lncRNAs in TSCC.

## 5. Conclusions

In this study, we have demonstrated and verified six immune-related lncRNAs that may collectively serve as an independent prediction model for the prognosis of TSCC. In addition, the model can be used to distinguish TSCC patients between high- and low-risk groups in TSCC patient population, which are known to have significantly different survival outcomes. To the best of our knowledge, this is the first study on the identification and construction of an immune-related lncRNAs prediction model for TSCC. The discovered six immune-related signature lncRNAs may provide further insight regarding both TSCC prognosis and development of immunotherapy strategies.

## Figures and Tables

**Table 1:** The demographics of the TSCC patients in this study.

Variable	Case (*n)*
Age	
≤53	48
>53	100
N/A	1
Gender	
Male	104
Female	45
T stage	
T1 + T2	68
T3 + T4	74
TX or N/A	7
Lymph Node Status	
N0	71
N1–3	70
NX or N/A	8
Metastasis	
M0	136
M1	1
MX or N/A	12
Stage	
Stage I + Stage II	49
Stage III + Stage IV	93
N/A	7

**Table 2:** Univariate Cox analysis identified 10 immune-related signature lncRNAs in the training cohort.

ID	Hazard Ratio	HR.95L	HR.95H	*p* Value
AC008147.2	1.618197807	1.159476115	2.258402832	0.0047
MIR4713HG	1.465332135	1.096913289	1.957491341	0.0097
AC009226.1	1.502288429	1.160577329	1.944610211	0.0020
AC079160.1	1.212107231	1.058402392	1.388133615	0.0054
AC104088.1	1.361078975	1.098591619	1.686282639	0.0048
LINC00534	1.322660403	1.108148844	1.578696356	0.0020
NAALADL2-AS2	1.243322929	1.075414330	1.437447748	0.0033
AC003092.1	1.336654339	1.078292842	1.656919858	0.0081
AC083967.1	1.650994013	1.349623551	2.019660393	<0.0001
FNDC1-IT1	1.662942115	1.171056335	2.361437613	0.0045

**Table 3:** Multivariate Cox analysis identified six immune-related signature lncRNAs in the training cohort.

ID	Coefficient	Hazard Ratio	HR.95L	HR.95H	*p* Value
MIR4713HG	0.4766	1.610596	1.128103	2.299451	0.0087
AC104088.1	0.3380	1.402013	1.104656	1.779414	0.0055
LINC00534	0.1640	1.178198	0.979253	1.417559	0.0082
NAALADL2-AS2	0.1566	1.169497	0.987941	1.384418	0.0069
AC083967.1	0.5811	1.787962	1.418558	2.253560	<0.0001
FNDC1-IT1	0.4401	1.552811	1.066420	2.261043	0.0217

## Data Availability

Data supporting this study are included within the article.

## References

[1] Almangush A., Heikkinen I., Mäkitie A.A., Coletta R.D., Läärä E., Leivo I., Salo T. (2017). Prognostic biomarkers for oral tongue squamous cell carcinoma: A systematic review and meta-analysis. Br. J. Cancer.

[2] Bello I.O., Soini Y., Salo T. (2010). Prognostic evaluation of oral tongue cancer: Means, markers and perspectives (I). Oral Oncol..

[3] Jeon J.-H., Kim M.G., Park J.Y., Lee J.H., Kim M.J., Myoung H., Choi S.W. (2017). Analysis of the outcome of young age tongue squamous cell carcinoma. Maxillofac. Plast. Reconstr. Surg..

[4] Garavello W., Spreafico R., Gaini R.M. (2007). Oral tongue cancer in young patients: A matched analysis. Oral Oncol..

[5] Vered M., Dayan D., Dobriyan A., Yahalom R., Shalmon B., Barshack I., Bedrin L., Talmi Y.P., Taicher S. (2010). Oral tongue squamous cell carcinoma: Recurrent disease is associated with histopathologic risk score and young age. J. Cancer Res. Clin. Oncol..

[6] Choi S.W., Moon E.K., Park J.Y., Jung K.W., Oh C.M., Kong H.J., Won Y.J. (2014). Trends in the incidence of and survival rates for oral cavity cancer in the Korean population. Oral Dis..

[7] Poling J.S., Ma X.J., Bui S., Luo Y., Li R., Koch W.M., Westra W.H. (2014). Human papillomavirus (HPV) status of non-tobacco related squamous cell carcinomas of the lateral tongue. Oral Oncol..

[8] National Cancer Institute Cancer Stat Facts: Tongue Cancer. https://seer.cancer.gov/statfacts/html/tongue.html.

[9] Almangush A., Bello I.O., Keski–Säntti H., Mäkinen L.K., Kauppila J.H., Pukkila M., Hagström J., Laranne J., Tommola S., Nieminen O. (2014). Depth of invasion, tumor budding, and worst pattern of invasion: Prognostic indicators in early-stage oral tongue cancer. Head Neck.

[10] Li G., Li X., Yang M., Xu L., Deng S., Ran L. (2017). Prediction of biomarkers of oral squamous cell carcinoma using microarray technology. Sci. Rep..

[11] Yang M., Xiong X., Chen L., Yang L., Li X. (2017). Identification and validation long non-coding RNAs of oral squamous cell carcinoma by bioinformatics method. Oncotarget.

[12] Statello L., Guo C.-J., Chen L.-L., Huarte M. (2021). Gene regulation by long non-coding RNAs and its biological functions. Nat. Rev. Mol. Cell Biol..

[13] Duncan L., Shay C., Teng Y. (2021). Multifaceted Roles of Long Non-Coding RNAs in Head and Neck Cancer. Adv. Exp. Med. Biol..

[14] Wu X., Gong Z., Ma L., Wang Q. (2021). lncRNA RPSAP52 induced the development of tongue squamous cell carcinomas via miR-423-5p/MYBL2. J. Cell. Mol. Med..

[15] Robinson M.D., McCarthy D.J., Smyth G.K. (2010). edgeR: A Bioconductor package for differential expression analysis of digital gene expression data. Bioinformatics.

[16] Mercer T.R., Dinger M.E., Mattick J.S. (2009). Long non-coding RNAs: Insights into functions. Nat. Rev. Genet..

[17] Rinn J.L., Chang H.Y. (2012). Genome Regulation by Long Noncoding RNAs. Annu. Rev. Biochem..

[18] Engreitz J.M., Haines J.E., Perez E.M., Munson G., Chen J., Kane M., McDonel P.E., Guttman M., Lander E.S. (2016). Local regulation of gene expression by lncRNA promoters, transcription and splicing. Nature.

[19] Salmena L., Poliseno L., Tay Y., Kats L., Pandolfi P.P. (2011). A ceRNA hypothesis: The Rosetta Stone of a hidden RNA language?. Cell.

[20] Huarte M. (2015). The emerging role of lncRNAs in cancer. Nat. Med..

[21] Gupta R.A., Shah N., Wang K.C., Kim J., Horlings H.M., Wong D.J., Tsai M.C., Hung T., Argani P., Rinn J.L. (2010). Long non-coding RNA HOTAIR reprograms chromatin state to promote cancer metastasis. Nature.

[22] Li H., An J., Wu M., Zheng Q., Gui X., Li T., Pu H., Lu D. (2015). LncRNA HOTAIR promotes human liver cancer stem cell malignant growth through downregulation of SETD2. Oncotarget.

[23] Ji P., Diederichs S., Wang W., Böing S., Metzger R., Schneider P.M., Tidow N., Brandt B., Buerger H., Bulk E. (2003). MALAT-1, a novel noncoding RNA, and thymosin beta4 predict metastasis and survival in early-stage non-small cell lung cancer. Oncogene.

[24] Wang Y., Chen F., Zhao M., Yang Z., Li J., Zhang S., Zhang W., Ye L., Zhang X. (2017). The long noncoding RNA HULC promotes liver cancer by increasing the expression of the HMGA2 oncogene via sequestration of the microRNA-186. J. Biol. Chem..

[25] Xiang J.F., Yin Q.F., Chen T., Zhang Y., Zhang X.O., Wu Z., Zhang S., Wang H.B., Ge J., Lu X. (2014). Human colorectal cancer-specific CCAT1-L lncRNA regulates long-range chromatin interactions at the MYC locus. Cell Res..

[26] Hung T., Wang Y., Lin M.F., Koegel A.K., Kotake Y., Grant G.D., Horlings H.M., Shah N., Umbricht C., Wang P. (2011). Extensive and coordinated transcription of noncoding RNAs within cell-cycle promoters. Nat. Genet..

[27] De Martino M., Esposito F., Pallante P. (2021). Long non-coding RNAs regulating multiple proliferative pathways in cancer cell. Transl. Cancer Res..

[28] Xie S.-J., Diao L.-T., Cai N., Zhang L.-T., Xiang S., Jia C.-C., Qiu D.-B., Liu C., Sun Y.-J., Lei H. (2021). mascRNA and its parent lncRNA MALAT1 promote proliferation and metastasis of hepatocellular carcinoma cells by activating ERK/MAPK signaling pathway. Cell Death Discov..

[29] Schmitt A.M., Chang H.Y. (2016). Long Noncoding RNAs in Cancer Pathways. Cancer Cell.

[30] Prensner J.R., Iyer M.K., Balbin O.A., Dhanasekaran S.M., Cao Q., Brenner J.C., Laxman B., Asangani I.A., Grasso C.S., Kominsky H.D. (2011). Transcriptome sequencing across a prostate cancer cohort identifies PCAT-1, an unannotated lincRNA implicated in disease progression. Nat. Biotechnol..

[31] Özeş A.R., Wang Y., Zong X., Fang F., Pilrose J., Nephew K.P. (2017). Therapeutic targeting using tumor specific peptides inhibits long non-coding RNA HOTAIR activity in ovarian and breast cancer. Sci. Rep..

[32] Xu F., Zhan X., Zheng X., Xu H., Li Y., Huang X., Lin L., Chen Y. (2020). A signature of immune-related gene pairs predicts oncologic outcomes and response to immunotherapy in lung adenocarcinoma. Genomics.

[33] Dai Y., Qiang W., Lin K., Gui Y., Lan X., Wang D. (2021). An immune-related gene signature for predicting survival and immunotherapy efficacy in hepatocellular carcinoma. Cancer Immunol. Immunother..

[34] Shen Y., Peng X., Shen C. (2020). Identification and validation of immune-related lncRNA prognostic signature for breast cancer. Genomics.

[35] Xu Q., Wang Y., Huang W. (2021). Identification of immune-related lncRNA signature for predicting immune checkpoint blockade and prognosis in hepatocellular carcinoma. Int. Immunopharmacol..

[36] Wang Y., Liu J., Ren F., Chu Y., Cui B. (2021). Identification and Validation of a Four-Long Non-Coding RNA Signature Associated with Immune Infiltration and Prognosis in Colon Cancer. Front. Genet..

[37] Tang C., Qu G., Xu Y., Yang G., Wang J., Xiang M. (2021). An immune-related lncRNA risk coefficient model to predict the outcomes in clear cell renal cell carcinoma. Aging.

[38] Miao T., Si Q., Wei Y., Fan R., Wang J., An X. (2020). Identification and validation of seven prognostic long non-coding RNAs in oral squamous cell carcinoma. Oncol. Lett..

[39] Jia B., Zheng X., Qiu X., Jiang X., Liu J., Huang Z., Xiang S., Chen G., Zhao J. (2021). Long non-coding RNA MIR4713HG aggravates malignant behaviors in oral tongue squamous cell carcinoma via binding with microRNA let-7c-5p. Int. J. Mol. Med..

[40] Fan Y., Zhou Y., Li X., Lou M., Gao Z., Tong J., Yuan K. (2022). Long Non-Coding RNA AL513318.2 as ceRNA Binding to hsa-miR-26a-5p Upregulates SLC6A8 Expression and Predicts Poor Prognosis in Non-Small Lung Cancer. Front. Oncol..

[41] Zhao X., Guo X., Jiao D., Zhu J., Xiao H., Yang Y., Zhao S., Zhang J., Jiao F., Liu Z. (2021). Analysis of the expression profile of serum exosomal lncRNA in breast cancer patients. Ann. Transl. Med..

[42] Hu J., Xu L., Shou T., Chen Q. (2019). Systematic analysis identifies three-lncRNA signature as a potentially prognostic biomarker for lung squamous cell carcinoma using bioinformatics strategy. Transl. Lung Cancer Res..

[43] Fan C.N., Ma L., Liu N. (2018). Systematic analysis of lncRNA-miRNA-mRNA competing endogenous RNA network identifies four-lncRNA signature as a prognostic biomarker for breast cancer. J. Transl. Med..

[44] Hu J., Wang T., Chen Q. (2019). Competitive endogenous RNA network identifies four long non-coding RNA signature as a candidate prognostic biomarker for lung adenocarcinoma. Transl Cancer Res.

[45] Boerrigter E., Benoist G.E., van Oort I.M., Verhaegh G.W., de Haan A.F.J., van Hooij O., Groen L., Smit F., Oving I.M., de Mol P. (2021). RNA Biomarkers as a Response Measure for Survival in Patients with Metastatic Castration-Resistant Prostate Cancer. Cancers.

[46] Groen L., Yurevych V., Ramu H., Chen J., Steenge L., Boer S., Kuiper R., Smit F.P., Verhaegh G.W., Mehra N. (2022). The Androgen Regulated lncRNA NAALADL2-AS2 Promotes Tumor Cell Survival in Prostate Cancer. Non-Coding RNA.

[47] Hong T., Parameswaran S., Donmez O.A., Miller D., Forney C., Lape M., Saint Just Ribeiro M., Liang J., Edsall L.E., Magnusen A.F. (2021). Epstein-Barr virus nuclear antigen 2 extensively rewires the human chromatin landscape at autoimmune risk loci. Genome Res..

